# A Challenging Case of Stent Dislodgement During Percutaneous Coronary Intervention Complicated by Peripheral Embolization

**DOI:** 10.7759/cureus.43212

**Published:** 2023-08-09

**Authors:** Frances Greathouse, Parth V Desai, Wajahat Humayun, Amir Darki

**Affiliations:** 1 Department of Internal Medicine, Loyola University Medical Center, Maywood, USA; 2 Department of Cardiology, Loyola University Medical Center, Maywood, USA

**Keywords:** small-balloon anchoring technique, interventional cardiology, catheterization, embolization, stent dislodgement

## Abstract

We report a challenging case of stent dislodgement for a 49-year-old male with a history of end-stage renal disease and insulin-dependent diabetes undergoing an elective coronary angiogram for cardiac risk stratification before kidney transplant surgery. A diagnostic transradial coronary angiogram was performed showing two severe type A lesions to the proximal and distal left circumflex artery (LCx). While attempting to stent the proximal LCx, the stent dislodged to the left main coronary artery (LMCA). The stent was successfully retrieved from the LMCA via the transradial route using the small balloon anchoring technique. Unfortunately, while attempting to retrieve the stent-balloon assembly, the stent was accidentally stripped off the balloon embolizing to the right superior gluteal artery. Given the stable location, no attempt was made to retrieve the stent and the patient had no complications on follow-up. This case highlights the challenges in managing coronary stent loss including risk factors for stent dislodgement, methods to retrieve the stent, and the risk of stent embolization.

## Introduction

Coronary stent dislodgement is an uncommon complication of percutaneous coronary intervention (PCI) [1]. It can lead to detrimental adverse effects if the stent is not retrieved from the coronary circulation, including significant bleeding, myocardial infarction, emergent coronary artery bypass graft (CABG), or death [2,3]. It is often caused by excessive coronary angulation, coronary calcification, tortuous lesions, or inadequate coronary artery predilatation [4]. Lost stents can be retrieved either percutaneously or surgically. There are multiple percutaneous methods for retrieval, with the most common being the small balloon anchoring and loop snare technique [2]. This case highlights the risk factors for stent dislodgement and the challenges with stent retrieval.

## Case presentation

**Figure 1:**
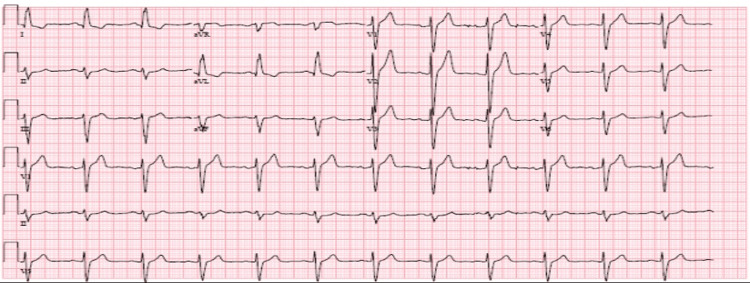
Preoperative electrocardiogram. This is the preoperative electrocardiogram demonstrating sinus rhythm with left axis deviation and left ventricular hypertrophy.

A 49-year-old male with a past medical history of end-stage renal disease (ESRD) on intermittent hemodialysis, obesity (body mass index of 38 kg/m^2^), insulin-dependent type 2 diabetes mellitus, and essential hypertension presented for an elective coronary angiogram for cardiac risk stratification before kidney transplant surgery. An electrocardiogram (EKG) and transthoracic echocardiogram (TTE) were completed as part of the preoperative workup. The EKG showed sinus rhythm with left axis deviation and left ventricular hypertrophy (Figure 1).

**Figure 2:**
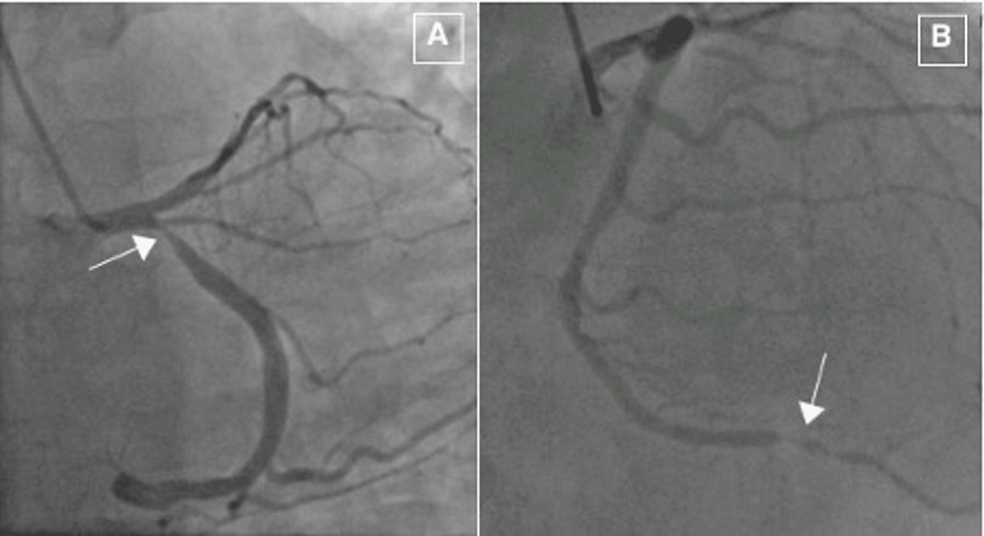
Left circumflex lesions. Severe type A proximal left circumflex (A) and distal left circumflex (B) lesions.

TTE showed a preserved left ventricular ejection fraction of 60-65% with normal left ventricle and right ventricle size and function. Additionally, a dobutamine stress test was completed which was inconclusive for ischemia due to failure to achieve the target heart rate. Given the patient’s history of insulin-dependent diabetes, ESRD, and nondiagnostic stress test, it was decided for the patient to undergo a coronary angiography for further risk stratification. A diagnostic transradial coronary angiogram was performed using a 6 Fr sheath which showed left dominant coronary circulation with two discrete severe (80%) type A lesions in the proximal and distal left circumflex artery (LCx) (Figure 2).

**Figure 3:**
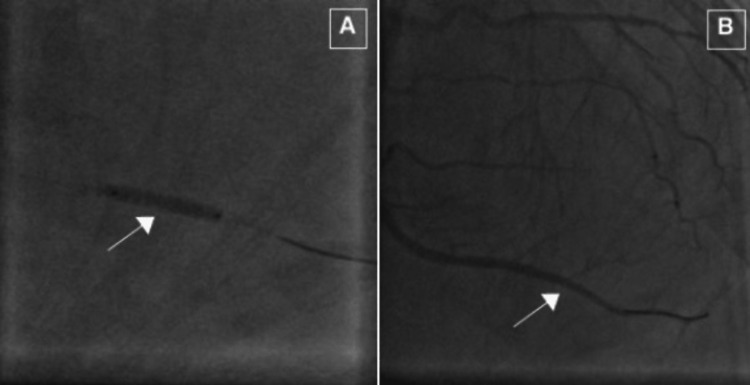
Successful distal left circumflex coronary artery percutaneous intervention. The distal lesion was crossed with a 0.014-inch Prowater Flex guidewire (A). Optical coherence tomograph-guided intervention of the distal lesion with an everolimus-eluting Xience Sierra stent 2.5 × 23 mm was performed with no residual stenosis (B).

The diagnostic catheter was exchanged with a 6 Fr EBU 3.0 guide catheter (Medtronic, Minneapolis, MN, USA) and the lesion was crossed with 0.014-inch Prowater Flex (Asahi Intecc USA, Irvine, CA, USA) guidewire with a successful intervention of the distal LCx lesion (Figure 3).

**Figure 4:**
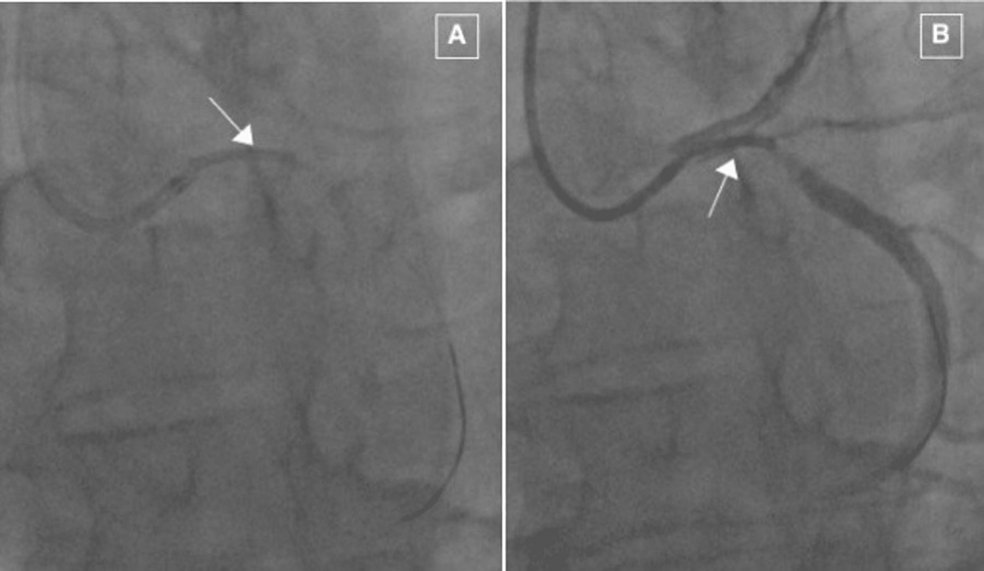
Stent dislodgement. Likely due to excessive angulation of the left circumflex artery, the stent was dislodged from the balloon while advancing for the proximal lesion (A). The stent was stranded over the guidewire at the left main coronary artery (B).

An attempt was made to deliver a 4.5 × 33 mm Everolimus-drug eluting Xience Sierra stent (Abbott Laboratories, Chicago, IL, USA) to the proximal LCx lesion, with predilatation applied before, but due to difficulty in delivering the stent, a 6 Fr guideliner support was used with a repeat attempt. Likely due to the excessive angulation of the LCx origin and the use of a larger stent, the stent dislodged from the balloon and was stranded over the guidewire at the left main coronary artery (LMCA) (Figure 4). No significant coronary calcification was identified on the optical coherence tomography (OCT), only a large lipid and fibrotic plaque.

**Figure 5:**
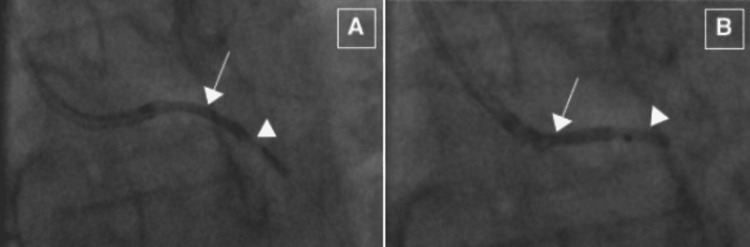
Stent retrieval using the small-balloon technique. A 1.5 mm × 15 mm Maverick balloon (arrow) catheter was placed over the guidewire and placed distal to the stent (arrowhead) (A). The balloon was inflated at 15 atm and the stent was carefully withdrawn outside of the left main coronary artery (B).

The stent balloon was exchanged with an anchoring balloon (1.5 mm × 15 mm Maverick Scimed, Boston Scientific, MN, USA) which was carefully placed distal to the stent and inflated at 15 atm. The stent was retrieved from the LMCA using the small-balloon anchoring technique, as described in prior studies [5], and kept at the tip of the guide catheter and moved to the distal aorta (Figure 5).

**Figure 6:**
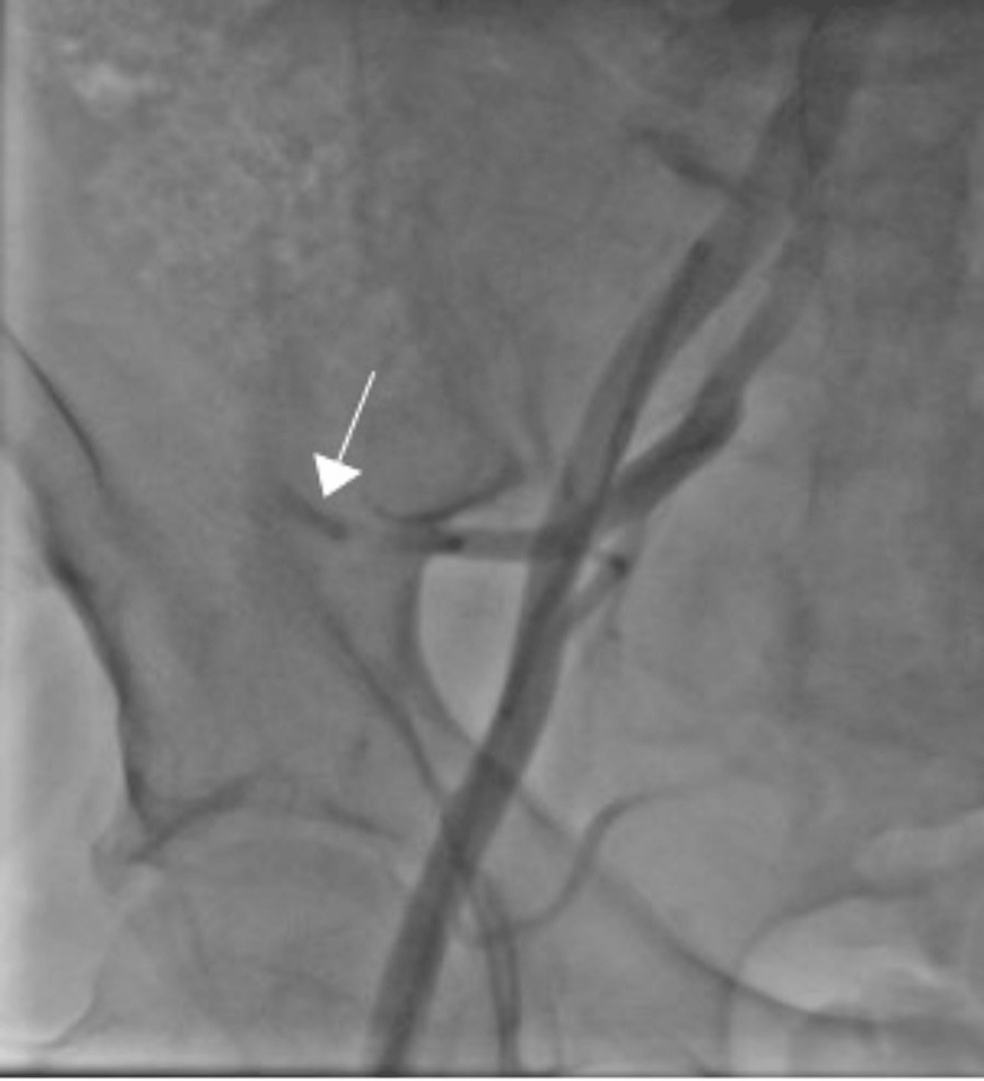
Embolization of the stent to the right superior gluteal artery. To retrieve the stent-balloon assembly, right femoral artery access was obtained. Using the goose-neck snaring method, it was attempted to snare the stent. However, the stent was stripped off the balloon and embolized to the right superior gluteal artery.

An attempt was made to retrieve the stent by introducing a goose-neck snare via an 8 Fr femoral artery sheath. Unfortunately, the stent was stripped off the balloon and was embolized which was found to be stably sitting in the right superior gluteal artery (Figure 6).

Given its stable position, no attempt was made to retrieve the embolized stent after discussing it with the vascular surgery team. Finally, the patient underwent successful OCT-guided PCI of the proximal LCx lesion with a 5.0 × 18 mm Everolimus-drug eluting Resolute Onyx stent with no residual stenosis. The total fluoroscopic time was 50 minutes and the total procedure time was 2 hours 30 minutes. The patient was observed in the hospital overnight. He was discharged the next day on dual antiplatelet therapy after ensuring he could ambulate without any symptoms. The patient followed up two weeks and six months after PCI with no evidence of right buttock or lower extremity claudication symptoms. The patient is currently active on the kidney transplant list and awaiting a donor kidney.

## Discussion

Coronary stent dislodgement is an uncommon complication of PCI with a reported incidence of 0.32-3.4% [1]. If no retrieval is achieved, stent loss can lead to severe complications, including myocardial infarction, emergent CABG procedure, or death due to compromised coronary blood flow or stent embolization [2]. In a systematic review and meta-analysis, most stents were removed uneventfully; however, some patients (one in five) experienced complications [3]. Therefore, it is critical to adequately retrieve the stent from the coronary circulation.

Stent loss occurs most frequently in patients with excessive angulation and coronary calcification [4]. As seen in our patient’s case, excessive angulation of the coronary vessel likely contributed to the stent dislodgement. Other risk factors include inadequate coronary artery predilatation and tortuous lesions [1]. To prevent stent loss during a PCI, it is recommended to avoid direct stenting, forceful advancement, and fast withdrawal of the stent delivery system. Proper vessel preparation with predilatation and the use of guide catheter extensions can also help decrease the risk of stent dislodgement in vessels with calcifications or excessive angulation [4].

Stent retrieval can be done either percutaneously or surgically. The transradial technique is preferred to the transfemoral technique due to a reduction in bleeding complications and 30-day mortality [6-8]. There are limitations to the transradial approach including the small size of the radial artery and the risk of spasm restricting the ability to upsize in sheath size [1]. Stent deployment/crushing can be considered in non-critical coronary segments where the stent is crushed against the coronary vessel wall. It should be avoided in critical coronary segments such as in the LMCA or bifurcation lesions. This route is not associated with an increased risk of cardiac complications and is often easier and faster [2]. The easiest technique in stent retrieval is the small-balloon anchoring technique (if the guidewire is through the stent), in which a small balloon is inserted through the stent, inflated at the distal end, and withdrawn. An alternative approach is to use a loop snare, advancing it through the guide catheter into the stent and snaring the stent for removal [2]. Other retrieval techniques that are not as commonly used include forceps, two wire technique, cook retained fragment retriever, and basket retrieval device [9].

If the percutaneous retrieval methods are not successful, then surgical options can be considered. Cha presented two case studies in which the stent was deformed, preventing removal through the radial artery sheath. Vascular surgery successfully retrieved the stent by radial artery dissection and repair [10]. In our patient’s case, the stent embolized to the peripheral circulation with no attempts to retrieve out of the body. The patient was followed two weeks and six months after PCI with no reported symptoms concerning for impaired blood flow to the gluteal artery. Therefore, it was decided the patient did not need additional imaging to access the gluteal artery blood flow. It has been reported that distal stent embolization usually has a benign clinical course and does not need removal. In a study by Alfonso et al., none of the eight patients with stent embolization had complications on the sixth-month follow-up [9,11].

## Conclusions

Stent dislodgement and embolization is an uncommon complication of PCI that can lead to serious adverse effects if retrieval is not achieved. This is an interesting case of stent loss in a patient with excessive angulation of the LCx. Although multiple retrieval techniques were used to retrieve the stent, unfortunately, the stent was stripped from the balloon and embolized to the right superior gluteal artery. It is important to understand the risk factors that increase the chance of stent dislodgement during PCI along with knowing the proper retrieval techniques to avoid serious complications.

## References

[1] Chikkabasavaiah NA, Raju SR, Jadav S, Rao SK, Nanjappa MC (2021). Device embolization during trans-radial percutaneous coronary intervention: various approaches - a case series. IHJ Cardiovasc Case Rep.

[2] Malik SA, Brilakis ES, Pompili V, Chatzizisis YS (2018). Lost and found: coronary stent retrieval and review of literature. Catheter Cardiovasc Interv.

[3] Alomar ME, Michael TT, Patel VG (2013). Stent loss and retrieval during percutaneous coronary interventions: a systematic review and meta-analysis. J Invasive Cardiol.

[4] Kostantinis S, Karacsonyi J, Simsek B, Brilakis ES (2022). Complications of stent loss during treatment of a heavily calcified and tortuous chronic total occlusion. Cardiovasc Revasc Med.

[5] Eggebrecht H, Haude M, von Birgelen C (2000). Nonsurgical retrieval of embolized coronary stents. Catheter Cardiovasc Interv.

[6] Jolly SS, Yusuf S, Cairns J (2011). Radial versus femoral access for coronary angiography and intervention in patients with acute coronary syndromes (RIVAL): a randomised, parallel group, multicentre trial. Lancet.

[7] Valgimigli M, Gagnor A, Calabró P (2015). Radial versus femoral access in patients with acute coronary syndromes undergoing invasive management: a randomised multicentre trial. Lancet.

[8] Romagnoli E, Biondi-Zoccai G, Sciahbasi A (2012). Radial versus femoral randomized investigation in ST-segment elevation acute coronary syndrome: the RIFLE-STEACS (Radial Versus Femoral Randomized Investigation in ST-Elevation Acute Coronary Syndrome) study. J Am Coll Cardiol.

[9] Brilakis ES, Best PJ, Elesber AA (2005). Incidence, retrieval methods, and outcomes of stent loss during percutaneous coronary intervention: a large single-center experience. Catheter Cardiovasc Interv.

[10] Cha KS (2012). Surgical retrieval of dislodged stent during transradial coronary intervention. J Invasive Cardiol.

[11] Alfonso F, Martinez D, Hernández R (1996). Stent embolization during intracoronary stenting. Am J Cardiol.

